# Supplementary material for: Protein Content and Amino Acid Profiles of Selected Edible Insect Species from the Democratic Republic of Congo Relevant for Transboundary Trade across Africa
Source: Insects. 2022 Oct 29;13(11):994. doi: 10.3390/insects13110994 (PMC9693131; doi:10.3390/insects13110994)
Supplement: Supplementary file 1 [file insects-13-00994-s001.zip › Figure S1.pdf]

SUPPLEMENTARY MATERIAL

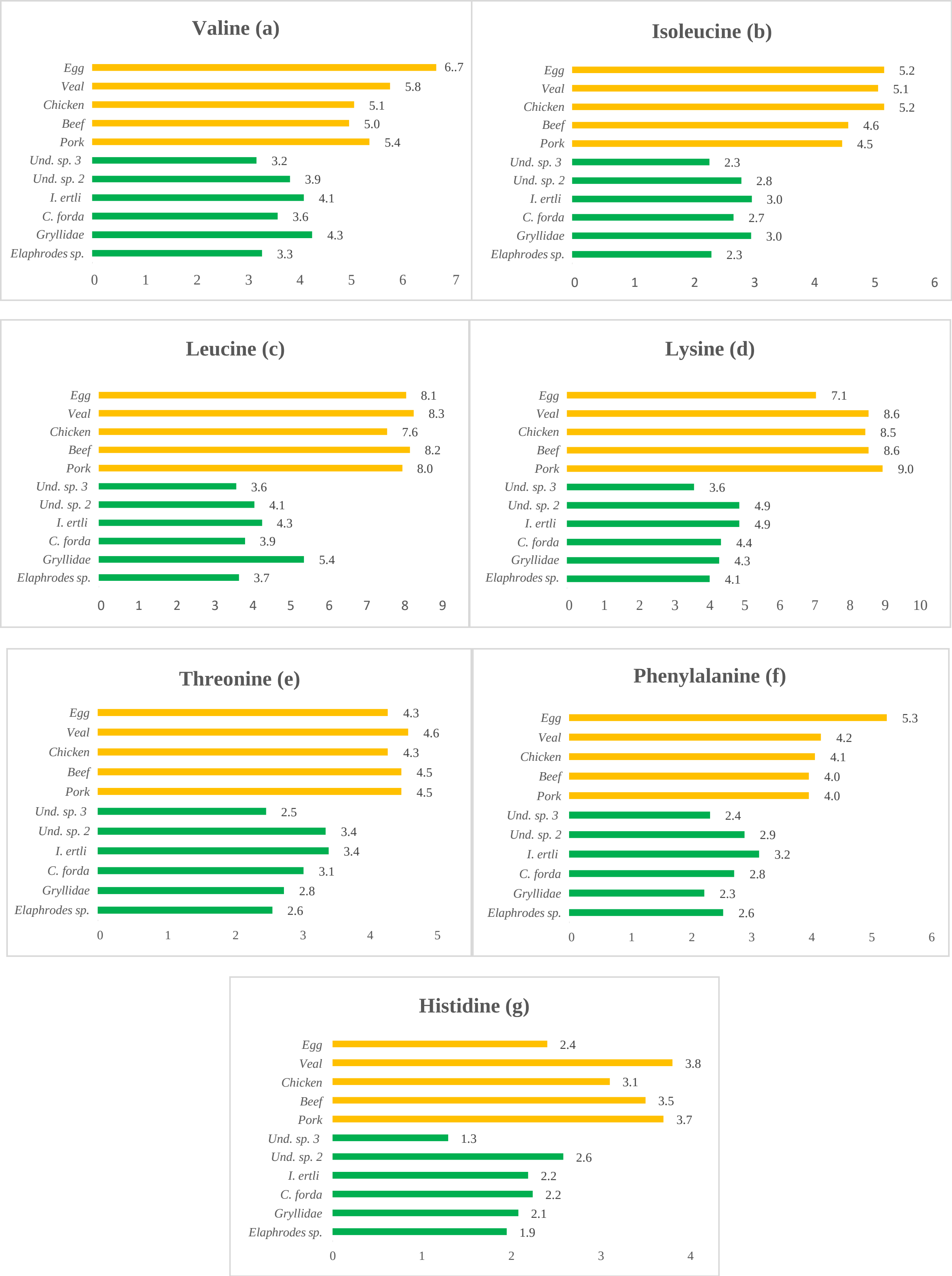

Figure S1 (a – g). Selected AA distribution's comparison in the protein fraction (% of total AA) of sampled insects with common sources of animal-based proteins - data other than insects are from Ghosh et al. [66].
