# Supplementary material for: Protein Content and Amino Acid Profiles of Selected Edible Insect Species from the Democratic Republic of Congo Relevant for Transboundary Trade across Africa
Source: Insects. 2022 Oct 29;13(11):994. doi: 10.3390/insects13110994 (PMC9693131; doi:10.3390/insects13110994)
Supplement: Supplementary file 1 [file insects-13-00994-s001.zip › Table S1 and S2.pdf]

SUPPLEMENTARY MATERIALS

Table S1. EAA profiles (g/100 g dry matter) of selected edible insects derived from literature

Edible insect species	His	Ile	Leu	Lys	Met + Cys	Phe + Tyr	Thr	Trp	Sources
**Lepidoptera**									
Saturniidae									
Cirina forda Westwood, 1849	2,080	3,680	5,910	4,590	1,280	8,450	5,190	1,840	[42]
Samia ricini Jones, W., 1791 (pupae)	2,670	4,420	6,630	6,540	2,840	14,640	4,750	N/A	[10,60]
Nudaurelia oyemensis Rougeot, 1955	1,810	2,560	8,270	7,980	4,320	13,430	4,450	1,600	[10,61]
Imbrasia truncata Aurivillius, 1909	1,740	2,420	7,310	7,890	3,870	13,870	4,690	1,650	[10,61]
Imbrasia ertli Rebel, 1904	N/A	3,600	3,670	3,930	2,920	3,060	4,050	0,810	[10,48]
Imbrasia epimethea Drury, 1773	1,970	2,860	8,100	7,420	4,110	14,000	4,800	1,600	[10,61]
Imbrasia obscura Butler, 1878	2,000	2,400	3,300	3,300	1,700	7,300	2,900	1,000	[62]
Notodontidae									
Anaphe venata Butler, 1878	0,780	2,140	1,310	0,880	N/A	4,640	0,380	0,000	[10,63]
**Orthoptera**									
Gryllidae									
Acheta domesticus (Linnaeus, 1758) (nymphs)	2,570	4,060	7,260	6,230	2,450	9,490	3,890	0,630	[10,64]
A. domesticus (adults)	2,340	4,590	10,000	5,370	2,290	8,050	3,610	0,630	[10,64]
A. domesticus (larvae)	2,100	4,200	7,300	5,600	3,600	7,400	3,500	0,600	[10,65]
Gryllodes sigillatus (Walker, 1869)	1,720	2,660	5,780	3,840	2,700	5,380	3,680	N/A	[66]
Gryllus bimaculatus De Geer, 1773	2,500	2,160	3,970	2,420	5,370	4,560	2,000	N/A	[67]
Gryllus assimilis (Fabricius, 1775)	2,520	3,360	6,620	5,290	3,430	7,600	3,090	2,530	[43]
Acrididae									
Schistocerca gregaria (Forskål, 1775)	2,060	2,820	7,770	3,510	1,180	5,180	3,550	N/A	[66]

Table S2. EAA profiles (mg/100 g fresh weight) of selected Saturniidae derived from literature

**Insect species**	His	Ile	Leu	Lys	Met + Cys	Phe + Tyr	Thr	Trp	Sources
Imbrasia truncata	666	809	1148	1449	563	3446	1036	245	[68]
Imbrasia epimethea	685	865	1250	1479	575	2938	1207	266	[68]
